# Supplementary material for: Enhanced IL-17 Producing and Maintained Cytolytic Effector Functions of Gut Mucosal CD161+CD8+ T Cells in SIV-Infected Rhesus Macaques
Source: Viruses. 2023 Sep 18;15(9):1944. doi: 10.3390/v15091944 (PMC10535321; doi:10.3390/v15091944)
Supplement: Supplementary file 1 [file viruses-15-01944-s001.zip › viruses-2595153-supplementary.pdf]

Supplementary Material

Enhanced IL-17 producing and maintained cytolytic effector functions of gut mucosal CD161$^+$CD8$^+$ T cells in SIV-infected rhesus macaques

Siva Thirugnanam[1], Edith M. Walker[1], Faith Schiro[1], Pyone P. Aye[1], Jay Rappaport[1], and Namita Rout[1, 2, 3*]

[1] Tulane National Primate Research Center, Covington, LA 70433, USA; tsivasakthivel@tulane.edu (S.T.); ewalker5@tulane.edu (E.M.W.); fcaillo@tulane.edu (F.S.); paye@tulane.edu (P.P.A.)
[2] Department of Microbiology and Immunology, Tulane University School of Medicine, New Orleans, LA 70112, USA
[3] Tulane Center for Aging, Tulane University School of Medicine, New Orleans, LA 70112, USA
* Correspondence: nrout@tulane.edu

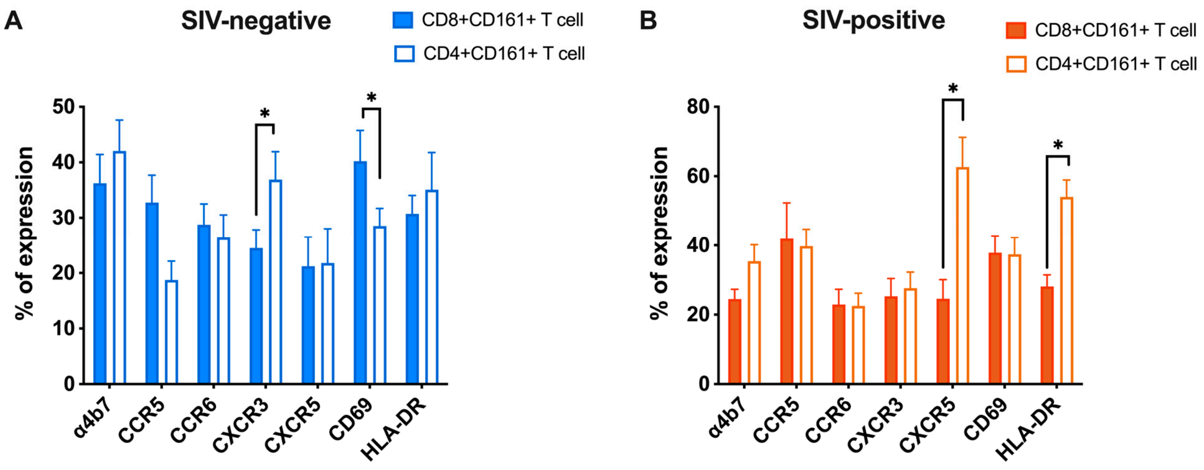

Figure S1. Difference between CD161⁺CD8⁺ T and CD161⁺CD4⁺ T cell expression of trafficking and activation markers within SIV-negative and SIV-positive groups. Comparison of frequencies of trafficking markers (α4β7, CCR5, CCR6, CXCR3, and CXCR5) and activation markers (CD69 and HLA-DR) between $CD161^{+}CD4^{+}$ T cells and $CD161^{+}CD8^{+}$ T cells within **(A)** SIIV-negative group and **(B)** SIV-infected group of macaques are shown for PBMC isolated from 13 SIV-negative macaques and 11 chronic SIV-infected macaques. Error bars show mean and SEM for each group. Groups were compared using Wilcoxon matched-pairs signed-rank test. Asterisks indicate significant differences between the groups (*$p < 0.05$).

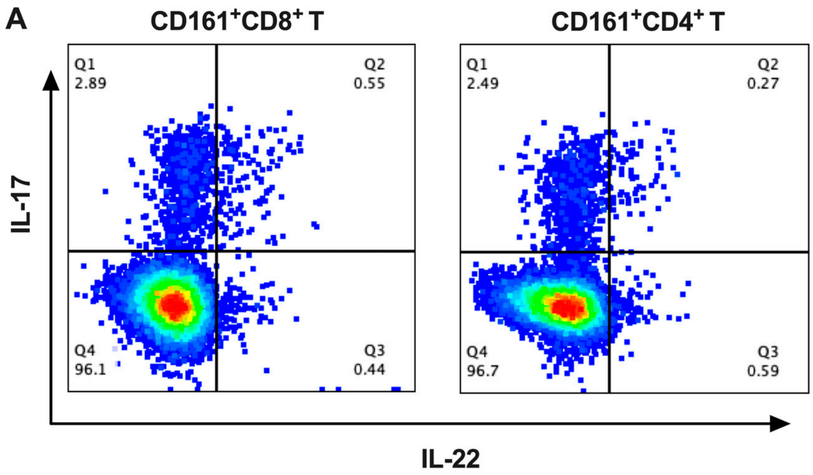

Figure S2. Flow cytometry plots showing CD161⁺ T cell subsets expressing IL-17 and/or IL-22 in a SIV-infected macaque. Intracellular cytokine staining for IL-17 and IL-22 cytokines was carried out on CD161-expressing T cells and evaluated by gating on $CD161^+$ $CD8^+$ T cells and $CD161^+CD4^+$ T cells.

Supplementary Table S1. Antibodies for Flow Cytometric Analysis.

Antibody	Flourochrome	Vendor	Clone	Catalog #
FVS700	AF700	BD		564997
CD45	BV510	BD	D058-1283	563530
CD3	APC-Cy7	BD	SP34-2	557757
CD4	BV650	BD	L200	563737
CD8	BV605	BD	SK1	564116
CD161	PE-Cy7	Biolegend	HP-3G10	339918
TCR g/d	PE-Cy7	Biolegend	B1	331222
TCR Va7.2	BV421	Biolegend	3C10	351716
CCR5/ CD195	APC	BD	3A9	560748
CCR6/ CD196	PCP-Cy5.5	BD	11A9	560467
CXCR3/ CD183	BV510	Biolegend	G025H7	353726
CXCR5	AL488	BD	RF8B2	558112
IL-18Rα	PE	R&D	70625	FAB840P
CD69	PE-CF594	BD	FN50	562617
Granzyme B	PE	BD	GB11	561142
IFN-γ	BV510	Biolegend	4S.B3	502544
IL-17	PCP-Cy5.5	eBioscience	eBio64DEC17	12-7179-42
IL-22	APC	Invitrogen	IL22JOP	17-7222-82
TNF-α	AF700	BD	MAb11	557996
CD1dTM	APC	*NIH Tetramer core*		
$\alpha_4\beta_7$	APC	*NHP Reagent resource*	A4B7R1	AB_2819257
